# Homicide by men diagnosed with schizophrenia: national case–control study

**DOI:** 10.1192/bjo.2020.129

**Published:** 2020-11-16

**Authors:** Alison Baird, Roger T. Webb, Isabelle M. Hunt, Louis Appleby, Jenny Shaw

**Affiliations:** Centre for Mental Health and Safety, University of Manchester, UK; Centre for Mental Health and Safety, University of Manchester; and NIHR Greater Manchester Patient Safety Translational Research Centre, UK; Centre for Mental Health and Safety, University of Manchester, UK; Centre for Mental Health and Safety, University of Manchester, UK; Centre for Mental Health and Safety, University of Manchester; and NIHR Greater Manchester Patient Safety Translational Research Centre, UK

**Keywords:** Homicide, schizophrenia, comorbidity, case-control, substance misuse

## Abstract

**Background:**

Some people diagnosed with schizophrenia are more prone to committing acts of serious violence, especially in the presence of drug or alcohol misuse. The rarity of homicide has meant that no large controlled study has previously examined clinical risk factors.

**Aims:**

To determine the risk factors for homicide by males diagnosed with schizophrenia.

**Method:**

A national nested case–control study of all previously admitted males diagnosed with schizophrenia, convicted of homicide between 1 January 1997 and 31 December 2012. Univariate and multivariable conditional logistic regression models were fitted to identify predictors of homicide in this population.

**Results:**

During the observation period 160 male patients with schizophrenia and a history of psychiatric admission were convicted of homicide, and they were matched with 542 male control patients who had not been convicted of homicide. Patients who committed homicide were more likely to have a history of violence and comorbid personality disorder or drug misuse. They were more likely to have missed their last contact with services prior to the offence and to have been non-adherent with their treatment plan. Almost all (94%) of homicides were committed by patients who had a history of alcohol or drug misuse and/or who were not in receipt of planned treatment.

**Conclusions:**

In England and Wales, homicides by patients with schizophrenia without substance misuse and in receipt of planned care are exceptionally rare. To prevent serious violence, mental health services should focus on drug and alcohol misuse, treatment adherence and maintaining contact with services.

The association between schizophrenia and violence has been widely researched,^1–4^ with a small subset of these studies focused on elevated homicide risk among individuals in this patient population.^5^ There are challenges in making comparisons between different published studies of links between schizophrenia and violence owing to the varying outcome definitions applied. Homicide committed by a person with schizophrenia is a rare event, with studies reporting that 6% of homicides in England are committed by these individuals.^6^ It is, however, important to examine the specific risk factors for homicide in this patient population, which is at elevated risk of committing homicide.^7^ In addition, the impact of homicide is significant owing to its grave effect on family and friends of the deceased.^8^ Flynn and colleagues found that a quarter of men convicted of homicide and over half of convicted female perpetrators in England and Wales had a lifetime history of mental illness and 10% had been in contact with mental health services in the year before the offence.^9^ Although little epidemiological evidence exists, studies have identified certain characteristics that are linked with elevated homicide risk among people with any diagnosed mental illness, including living alone, being unemployed,^10^ alcohol misuse,^5^ substance misuse,^3^ presence of specific disease symptoms (such as delusions and hallucinations^11^), a change in the nature or magnitude of the person's delusional beliefs,^12^ longer duration of untreated illness^9^ and non-adherence to medication.^13^ Wang and colleagues conducted a large population-based case series of homicide offenders (*n* = 669) diagnosed with schizophrenia in Hunan Province, China, to examine gender differences.^14^ However, these were case series studies that reported descriptive findings and causality cannot be inferred from them. Few controlled studies of homicide committed by people diagnosed with schizophrenia have been published. A national case–control study conducted in Sweden reported an increased risk of homicide after discharge from in-patient care among people with psychosis who had a history of drug and alcohol misuse prior to and following admission, and also in those who were non-adherent with their medication following discharge.^7^ However, statistical power and precision were low because of the small number of homicide cases ascertained even in this national registry study. To enhance power the case definition in the study also included convictions for attempted homicide. In addition, the authors indicated that, as theirs was a study conducted using routinely collected registry data, they were limited in the narrow range of potential risk factors that could be examined; they could not, for instance, examine the potential importance of comorbid personality disorder.

Homicide perpetrated by people with schizophrenia is an important topic that has not been extensively examined using robust epidemiological study designs. This dearth of evidence is partly due to the exceptional rarity of homicide as an outcome, which presents major challenges for researchers. To our knowledge, this is the first national case–control study of homicide by people diagnosed with schizophrenia to be conducted in the UK. On the basis of previously reported research findings we hypothesised that the risk factors for homicide by people with schizophrenia would be similar to those reported for all forms of violent criminality in this population.^3,5,7,10–14^ These include social factors such as living alone and unemployment, and clinical considerations such as substance misuse, non-adherence to treatment programmes and missed appointments with mental health services.

## Method

### Delineation of the nested case–control study

The case patients constituted a complete national case series of 160 males convicted of committing homicide in England and Wales between 1 January 1997 and 31 December 2012, who were diagnosed with schizophrenia (schizophrenia, schizotypal and delusional disorders as recorded by the mental health team completing the study questionnaire) and were in contact with mental health services in the year prior to the index homicide offence; they also had a history of admission to in-patient care, but were not in-patients at the time of the offence. The study excluded individuals with schizophrenia who had no history of in-patient admission, or had no previous contact with mental health services, because comparable control data were unavailable for this group. Data on the case patients from England and Wales were extracted from the National Confidential Inquiry into Suicide and Safety in Mental Health (NCISH) (formerly the National Confidential Inquiry into Suicide and Homicide by People with Mental Illness). The NCISH holds a complete UK-wide consecutive case series of all ‘patient homicides’, i.e. individuals who were convicted of homicide since April 1996 who had been in recent contact with mental health services.^15^ It collects detailed clinical information such as the number of perpetrators with a history of mental illness, their clinical and psychosocial characteristics, and the circumstances in which each homicide occurred (including the relationship of the victim and perpetrator and methods of killing). The NCISH captures information on people convicted of murder, manslaughter or infanticide, as well as individuals who received verdicts of not guilty by reason of insanity or were deemed unfit to plead.

Data pertaining to control patients were obtained from Hospital Episode Statistics (HES), a data-set managed by the Health and Social Care Information Centre (HSCIC) that contains details of in-patient, out-patient and accident & emergency department records for all National Health Service (NHS) hospitals in England. The data are stored as individual patient records with one record per episode of care. Data were obtained on all male patients with a diagnosis of schizophrenia or delusional disorder who were discharged from in-patient care between 1 January 1997 and 31 December 2012. Equivalent data on patients who had out-patient/community contact with services were only available from 2003. We therefore included only case and control patients with a history of in-patient admission; case patients without a history of admission (*n* < 5 persons) were removed from the data-set. Control patients were randomly selected and individually matched to case patients on age using year of birth as the matching criterion. Matching on year of birth took account of cohort effects as well as the potential confounding influence of age. We aimed to match a ratio of 1 homicide case to 5 control patients where possible to maximise statistical power, as determined by the number of homicide perpetrators within the national case series.^16^ Greater power would not be gained by increasing the case–control ratio beyond 5. Matching of 1:5 was possible for 24% of all case patients but for most case–control matches this ratio was lower than 5. A 1:4 matching was achieved for 23%, 1:3 for 21%, 1:2 for 19% and the remaining 13% were matched on a ratio of 1:1. To be eligible for selection as a control, patients must have been in contact with mental health services in the year prior to the matched case patient's index offence, and must not have committed a homicide prior to it. A total of 542 males diagnosed with schizophrenia and with a prior episode of psychiatric in-patient care were selected as matched control patients.

### Data collection procedures

During the study period the NCISH was informed annually of all perpetrators who were convicted of homicide (murder, manslaughter and infanticide) from the Homicide Index at the Home Office. Information on previous convictions for all homicide perpetrators nationally was then collected from the Police National Computer database, which was accessed via Greater Manchester Police. The conviction data also provided details of the perpetrator's address at the time of the index offence. We then contacted the pertinent NHS trust in the area where the person lived to establish whether or not they had been in contact during the 1-year period preceding the index offence.^15^ For those individuals who were in recent contact with services, a questionnaire was sent to the consultant psychiatrist who was responsible for their care to glean further detailed information on their sociodemographic and clinical characteristics, and the treatment that they had received. The questionnaire collects information on the patient's demographics, psychosocial history, previous violence, treatment and adherence to treatment, the last in-patient admission prior to the offence, recent contact with services, including missed appointments, risk of violence and symptoms of mental illness at last contact.

From the HES data-set, patient ID number, NHS number, date of birth, admission date, discharge date, treatment provider code and consultant code were extracted for each control patient. This information enabled us to identify the NHS trust and consultant psychiatrist responsible for the patient's care during the in-patient episode. If the consultant was no longer employed by the trust or could not be identified using the General Medical Council's website, we contacted the trust's medical director to request that they nominate an alternative member of staff to complete the questionnaire. This was often a senior member of the mental health team. We sent a detailed questionnaire to the clinician to complete regarding the treatment received by the control patient prior to the matched case patient's offence date. This date is hereafter termed the ‘index date’. The tailored study questionnaire was based on the standard NCISH homicide questionnaire, to collect equivalent information, but with all references specifically referring to an offence of homicide omitted, instead referring to ‘index date’. As with the NCISH questionnaire, the survey requested information concerning the patient's sociodemographic characteristics, their clinical and forensic histories, and aspects of their care and treatment received.

As we were unable to obtain the names of the control patients, we could not request Police National Computer (PNC) data on previous convictions. The information on violence prior to homicide was taken from the questionnaire for case and control patients and was based on clinicians’ knowledge of previous violent episodes, and this variable was not subdivided by specific offence type in the NCISH data-set.

### Diagnoses

Primary and secondary diagnoses were recorded by the mental health team completing the questionnaire, based on ICD-10 guidelines. HES provided data on patients with a diagnosis of schizophrenia or delusional disorder. HES obtain information on diagnosis from the hospital records of the in-patient admission. This diagnosis was also confirmed with the mental health team completing the control questionnaire. Owing to unavailability of this variable in the NCISH data-set, subdivision according to specific disorders in the schizophrenia spectrum was not possible.

### Area-level deprivation score

This ecological measure was obtained by linking the last known postcode of residence for each homicide perpetrator to the 2010 Index of Multiple Deprivation (IMD) for England.^17^ The IMD is derived via a weighted combination of the following seven domains of deprivation: income; employment; health and disability; education, skills and training; barriers to housing and services; living environment; and crime. Each postcode was linked to a deprivation score via lower super output areas (LSOAs) using the online geographical matching tool GeoConvert.^18^ Residential postcode data were available for 85% of the 160 homicide perpetrators in the national case series. Equivalent information was unavailable for the control patients as we were unable to obtain the names and addresses of these individuals.

### Statistical analysis

Data were analysed using STATA 11 software for Windows.^19^ Relative risks were estimated as exposure odds ratios generated from conditional logistic regression models, with multivariable models fitted using a backwards elimination approach to identify mutually independent predictors of homicide among patients diagnosed with schizophrenia. Statistical significance was set at 5% (two-sided) for all analyses.

### Ethics statement and role of the study sponsor

The authors assert that all procedures contributing to this work comply with the ethical standards of the relevant national and institutional committees on human experimentation and with the Helsinki Declaration of 1975, as revised in 2008. All procedures involving human patients were approved by the NHS Health Research Authority, National Research Ethics Service (NRES) Committee North West, Haydock (REC reference: 11/NW/0614). Exemption under section 251 of the National Health Service Act 2006, enabling access to confidential and identifiable information without informed consent in the interest of improving care, was therefore also obtained from the Health Research Authority Confidentiality Advisory Group (HRA-CAG).

The study's sponsor, the University of Manchester, has insurance policies in place to cover design, management, and conduct of the research.

## Results

Between 1997 and 2012, 160 male patients diagnosed with schizophrenia and with at least one previous hospital admission committed homicide. The median age at the time of the offence was 33.5 years (interquartile range IQR = 15). Just over half of all victims were male (Table 1) and the median age of the victims was 44 years (IQR = 29). Victims were most commonly family members, followed by acquaintances. The most frequent method of killing was by sharp instrument. Despite all perpetrators having been diagnosed with schizophrenia and having had contact with mental health services prior to the offence, just over a quarter were convicted of murder and one-third were sentenced to prison.

**Table 1:** Characteristics of the homicide offences (*n* = 160)

Characteristics	*n*	%
Male victim	87	54%
Victim was a stranger	19	14%
Victim was an acquaintance	47	34%
Victim was a current/former spouse/partner	23	17%
Victim was a family member	48	35%
Method of homicide: sharp instrument	100	65%
Court verdict		
Murder	43	27%
Insane/unfit to plead	8	5%
Manslaughter by diminished responsibility^a^	59	37%
Other manslaughter	50	31%
Prison	53	33%
Hospital order	105	67%

a. Under section 2 of the Homicide Act 1957.

Univariate conditional logistic regression analyses of psychosocial and clinical characteristics and clinical care for case versus control patients are shown in Table 2. Compared with control patients, homicide perpetrators were more likely to belong to Black, Asian and minority ethnic (BAME) groups, to have histories of violence or drug misuse documented in their case notes, to have a secondary diagnosis of personality disorder or drug dependence/misuse, to have missed their last appointment with mental health services prior to the offence, and to be non-adherent with their medication. Perpetrators were less likely than control patients to have been recently discharged and to have a follow-up appointment scheduled after release from in-patient care. The prescribing of antipsychotics and a routine last contact prior to the index date were more common among control patients.

**Table 2:** Univariate conditional logistic regression models indicating factors associated with higher or lower homicide risk among patients with schizophrenia

	Case patients (*n* = 160)		Control patients (*n* = 542)			
	*n*	%	*n*	%	OR (95% CI)	*P*
Sociodemographic factors						
Unmarried	134	84%	476	88%	0.37 (0.20–0.69)	**0.001**
Unemployed	104	66%	347	64%	0.90 (0.60–1.35)	0.61
Living alone	72	45%	272	50%	0.70 (0.48–1.02)	0.06
Black, Asian and minority ethnic (BAME) group	53	34%	125	23%	1.83 (1.22–2.74)	**0.004**
Clinical factors						
History of alcohol misuse	87	54%	241	44%	1.47 (1.02–2.12)	**0.04**
History of drug misuse	112	70%	292	54%	2.09 (1.37–3.20)	**0.001**
History of violence	110	69%	200	37%	4.18 (2.74–6.37)	**<0.001**
History of self-harm	55	34%	199	37%	0.86 (0.57–1.27)	0.44
Any secondary diagnoses	98	61%	218	40%	2.16 (1.49–3.12)	**<0.001**
Discharged from in-patient care within 3 months prior to the offence/index^a^	23	14%	117	22%	0.53 (0.32–0.89)	**0.02**
Missed last appointment with services prior to the offence/index	60	38%	84	16%	2.92 (1.95–4.37)	**<0.001**
Secondary diagnosis prior to offence						
Personality disorder	26	16%	23	4%	5.01 (2.61–9.62)	**<0.001**
Alcohol dependence/misuse	26	16%	67	12%	1.24 (0.74–2.07)	0.41
Drug dependence/misuse	69	43%	116	21%	2.91 (1.92–4.42)	**<0.001**
Affective disorder	14	9%	24	4%	1.77 (0.88–3.55)	0.11
History of illness <12 months	16	10%	33	6%	1.55 (0.82–2.96)	0.18
History of illness >5 years	97	61%	336	62%	1.02 (0.65–1.61)	0.91
Over 5 previous admissions	28	18%	133	25%	0.65 (0.40–1.06)	0.08
Prescribed antipsychotics	129	89%	494	98%	0.14 (0.06–0.35)	**<0.001**
Prescribed lithium	10	7%	52	10%	0.65 (0.32–1.34)	0.24
Prescribed SSRI	19	13%	77	14%	0.79 (0.45–1.40)	0.42
Non-adherent with medication	50	31%	86	16%	2.41 (1.57–3.70)	**<0.001**
Distressing psychotropic drug side-effects	16	10%	65	12%	0.87 (0.48–1.59)	0.66
Last admission was under the MHA	82	51%	238	44%	1.35 (0.93–1.97)	0.11
Follow-up appointment was made following discharge	127	80%	458	85%	0.18 (0.04–0.79)	**0.02**
Last admission was for <1 week	14	9%	47	9%	1.07 (0.58–2.00)	0.82
Last admission was for >13 weeks	41	26%	135	25%	0.92 (0.60–1.42)	0.72
Patient initiated discharge	17	11%	31	6%	2.01 (1.07–3.79)	**0.03**
Last contact with services						
Last contact within 7 days of index	53	33%	157	29%	1.06 (0.71–1.57)	0.78
Last contact was routine	112	70%	434	80%	0.30 (0.19–0.48)	**<0.001**
Symptoms of mental illness	64	40%	200	37%	0.93 (0.63–1.38)	0.71
Delusions/hallucinations	28	18%	135	25%	0.54 (0.34–0.87)	**0.01**
Consultant completing the questionnaire had been patient's RMO prior to the index	88	55%	246	45%	1.38 (0.96–1.99)	0.08

SSRI, selective serotonin reuptake inhibitor; MHA, Mental Health Act 1983; RMO, responsible medical officer.

a. The ‘index’ is the date of the matched case patient's offence.

Bold values denote statistical significance at the *P* < 0.05 level.

The final multivariable model presented in Table 3 indicates that elevated homicide risk was independently associated with missed appointments with services, being from a BAME group, having a history of violent criminality, and having a secondary diagnosis of personality disorder or drug dependence/misuse. Recent discharge from in-patient care, multiple admissions, routine contact, the prescribing of antipsychotics and the presence of delusions/hallucinations at last contact were independently associated with lower homicide risk.

**Table 3:** Final multivariate conditional logistic regression model indicating mutually adjusted independent risk and protective factors for homicide among patients with schizophrenia

	OR (95% CI)	*P*
Risk factors		
Missed last appointment with mental health services	1.93 (1.08–3.45)	0.03
Black, Asian and minority ethnic (BAME) group	1.85 (1.04–3.26)	0.04
Previous violence documented in case notes	4.24 (2.44–7.35)	<0.001
Secondary diagnosis of personality disorder prior to offence	2.98 (1.23–7.21)	0.02
Secondary diagnosis of drug dependence/misuse	2.34 (1.34–4.09)	<0.01
Protective factors		
Discharged from in-patient care within 3 months prior to the offence/index^a^	0.45 (0.22–0.91)	0.03
Over 5 previous admissions	0.43 (0.22–0.84)	0.01
Prescribed antipsychotics	0.19 (0.07–0.56)	<0.01
Last contact was routine	0.18 (0.09–0.36)	<0.001
Delusions/hallucinations at last contact	0.33 (0.17–0.66)	<0.01

a. The ‘index’ is the date of the matched case patient's offence.

**Fig. 1:**
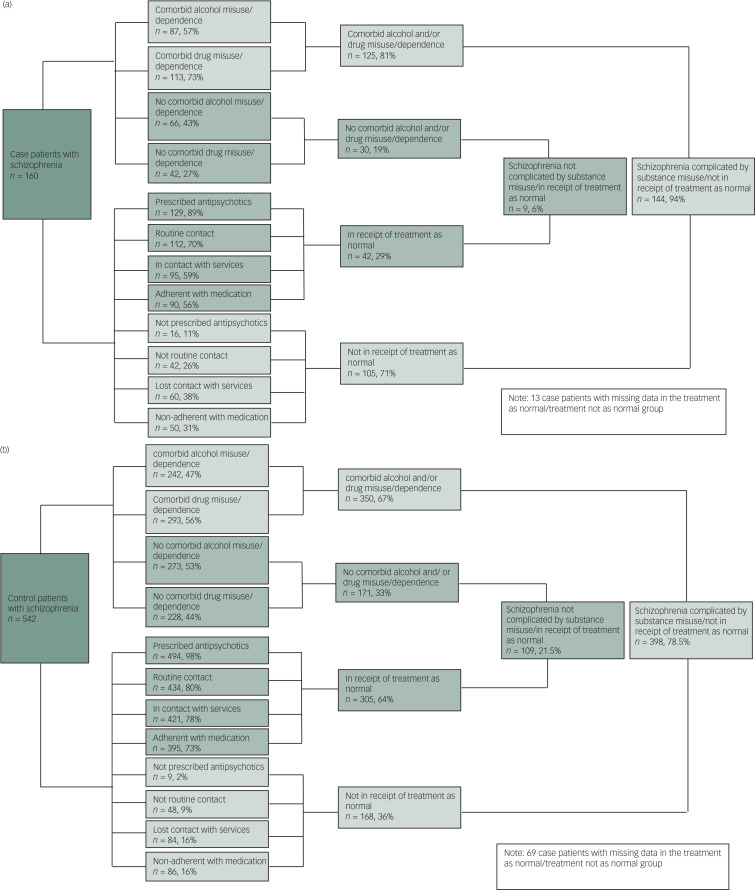
(a) Quantification of components of schizophrenia among patients convicted of homicide (cases). (b) Quantification of components of schizophrenia among patients who did not commit homicide (controls).

Figure 1 shows the proportions of case and control patients who have schizophrenia complicated by other risk factors (comorbid substance misuse/dependence and/or not in receipt of standard treatment for schizophrenia) compared with those without comorbid substance misuse and who are receiving standard treatment. Among both case and control patients the proportion of individuals with schizophrenia complicated by substance misuse and non-treatment receipt was far greater than the proportion of other patients. However, when comparing the two groups, proportionally more patients who were convicted of homicide had schizophrenia complicated by substance misuse and non-treatment compared with controls, with the disparity between case and control patients being especially pronounced in relation to the proportion not being in receipt of treatment as planned (71% of cases versus 36% of controls, *P* < 0.001). Personality disorder was extremely rare (*n* < 3) in case and control patients who were in receipt of treatment as normal and/or did not have comorbid substance misuse. Among patients who were not in receipt of standard treatment and had comorbid substance misuse, personality disorder was more common in case patients when compared with control patients (*n* = 25, 17% *v*. *n* = 21, 5%, *P* < 0.001). Just nine patients (i.e. 6%) among the case series of 160 perpetrators had no history of alcohol or drug misuse and were receiving standard treatment and care at the time of the index offence.

### Socioeconomic status

Previous studies have reported a link between lower socioeconomic status and increased violence risk in this patient population.^18^ We examined the distribution of deprivation scores among the homicide perpetrators, but could not do likewise for the control patients as we were not granted permission to access their address details on the date when the index offence occurred. Almost three-quarters (41/57; 72%; 95% CI 59–82%) of BAME perpetrators residing in England lived in the three highest deprivation deciles, compared with just over a half (54/99; 55%; 95% CI 45–64%) of White perpetrators, and this difference was statistically significant (*z* = 2.1, *P* = 0.03).

## Discussion

### Summary of main findings

In this national nested case–control study of previously admitted male patients diagnosed with schizophrenia, those who were convicted of homicide were more likely to be non-adherent with their treatment plan, to have lost contact with services prior to the offence, to have a history of violent criminality, to have a comorbid personality disorder or drug use disorder, to have been admitted multiple times, or to belong to a BAME group. Homicide perpetrators were less likely to have had recent routine contact with services and to have been recently discharged from hospital. Some observed differences between cases and controls, including the outcome (homicide), were attributable to differences in treatment. These findings suggest that much of the risk of serious violence in schizophrenia is related to comorbidity, and that maintaining satisfactory levels of care and follow-up may be linked with reduced risk of serious violence. Almost all (94%) of those who committed homicide either had a history of alcohol or drug misuse or had not received treatment and care as planned. Among the 160 homicides committed by this patient group during the 15-year observation period, only 9 occurred in the absence of these clinical features – fewer than 1 per year.

### Comparison with existing evidence and interpretation

From their systematic review and meta-analysis, Large and colleagues concluded that future research on homicide perpetrators in this patient population should focus on comorbid drug and alcohol misuse and on individuals of lower socioeconomic status.^20^ In the national case series, the majority of homicide perpetrators resided in the most deprived localities in England. Increased prevalence of violence and greater availability of weapons and illicit substances in poorer areas may have a disproportionate impact on people diagnosed with schizophrenia, who may be more susceptible to the harmful influences of area-level and/or individual-level sociodemographic risk factors.^20^

An earlier published report expressed concerns regarding the capacity of mental health services to provide treatment for persons diagnosed with a personality disorder and/or substance misuse, particularly given the preponderance of adverse outcomes (violence and self-harm) in these patient groups.^22^ Studies have concluded that specialist mental health services for people with personality disorder are improving despite the evidence-base for treatment being limited.^23,24^ We found that a secondary diagnosis of personality disorder or drug misuse/dependence was associated with elevated homicide risk among men diagnosed with schizophrenia. In 2012, addiction services in England were transferred from the control of NHS services to local authorities, having previously been jointly commissioned.^25^ The move resulted in concern among mental health professionals regarding the suitability of the newly commissioned services, including their capacity to respond adequately to the mental health needs of patients, lack of continuity of care, limited collaboration between services and delays in treatment following referral.^25,26^ In addition there has been unease that the re-tendering processes for services every 3 years results in a lack of a consistent care provider for patients and staff and that funding restrictions placed on organisations competing to tender for these short-term contracts result in addiction specialists being replaced with less medically qualified staff.^25,26^ There is a need for more collaboration and joint working between mental health and substance misuse services, including development and evaluation of new models of care.

Previously published analyses of the NCISH case series revealed that 59% of homicide perpetrators diagnosed with schizophrenia by the consultant preparing a court report were experiencing delusions at the time of the offence,^11^ and just over half reported a change in the nature of their delusions in the month preceding the homicide. We found that 18% were experiencing delusions and/or hallucinations at the time of last contact prior to the index offence, a figure considerably lower than previously reported.^11^ This discrepancy may have arisen because a number of perpetrators were asymptomatic at the time of last contact and developed delusions and hallucinations after their last contact with services. This would have subsequently been discovered following the offence by the expert witness undertaking the psychiatric examination in preparing a court report.

To our knowledge there has been only one previously published case–control study of homicide perpetration by recently discharged patients with psychosis.^7^ The study examined medical records of 47 individuals with a diagnosis of psychosis (schizophrenia, bipolar disorder or other psychotic illnesses) who committed homicide or attempted homicide within 6 months of discharge from in-patient care in Sweden between 1988 and 2001 and those of 105 matched control patients of the same diagnostic subgroup who had did not perpetrate homicide and had no convictions for violence subsequent to admission. Consistent with our findings, Fazel and colleagues also found that substance misuse and non-adherence with medication were associated with elevated homicide risk.

### Strengths and limitations

A number of studies have identified potential risk factors for schizophrenia and violence, including homicide.^3–5,7,20,27,28^ Our 16-year national investigation of homicide by people with schizophrenia is the only published case–control study of homicide risk among persons diagnosed with schizophrenia to have been conducted in the UK. The NCISH database is a large national homicide case series and contains robust representative clinical data and its methodology has established validity.^29^ The NCISH questionnaire data are collected from patients’ supervising clinicians and has the benefit of being based on clinical opinion, which is supplemented with information from patients’ case notes. This is the unique strength of the NCISH case series, as such a wealth of information does not exist in other databases, including national administrative registers. As we examined data on patients diagnosed with schizophrenia who committed homicide, and on equivalent age-matched control patients with schizophrenia who did not perpetrate homicide, we could make comparisons between the two groups and confirm previously indicated risk and protective factors. We received a return rate of 80% for control questionnaires and 95% for NCISH homicide questionnaires.

Some key limitations, however, should be acknowledged. The study was conducted using the NCISH case series for England and Wales, and findings may therefore have potentially limited generalisability beyond those countries. Data were available only for convicted homicide offenders and we therefore could not examine persons who had committed serious non-fatal violent crimes in this study. The study shows that there are more risk factors among men with schizophrenia who commit homicide than those who do not. It may be that these are related to all seriously violent men and not specific to violent men with schizophrenia. In addition, as we did not compare our case patients who committed homicide with a sample of all homicide offenders, we cannot be sure that these risk factors do not apply to homicide offenders without a diagnosis of schizophrenia. We did not have information about the role of schizophrenia in the homicide, as this was beyond the scope of the study. It must be noted that despite a diagnosis of schizophrenia, 27% in the case group were convicted of murder but we do not know whether diminished responsibility was raised as a defence and, if it was, whether this was considered. Clinical information was collected retrospectively based on the account of the clinician involved. Diagnoses were made by clinical teams using ICD-10 criteria and not standardised interviews, and explanatory variables were derived using information captured in patients’ clinical records rather than by application of validated risk assessment tools. However, clinicians completing the questionnaire used both case notes and personal knowledge of the patient to provide information. As the data were retrospectively obtained, the information provided by clinicians may have been biased by their awareness of outcome status. For example, consultants may have recalled exposures differently for the homicide perpetrators than for the control patients, and inaccuracies in the data collected may have led to either underestimation or overestimation of exposure prevalence values. Ideally, to minimise recall bias consultants should be masked to outcome status, i.e. whether the patient had committed a homicide or not. However, owing to the disturbing nature of this phenomenon and subsequent newspaper coverage, expert witness requests and independent investigations, it may be that consistent masking to outcome status is implausible. Furthermore, it may be that some participating consultants had discerned the factors that we had hypothesised would be associated with higher or lower homicide risk; for example, history of violence, comorbidity, loss to follow-up or non-adherence with medication. The responses that these consultants provided to these questions may also have been influenced by their prior knowledge of the patient in question and of the candidate risk factors. Observer bias of this nature is a limitation of many retrospective observational studies of homicide.^30^ However, the majority of the questions in the questionnaire were objective, which minimised the need for treating consultants to make subjective judgements when completing these items. Certain clinical care measures are likely to be linked. For example, both treatment adherence and loss of contact were strongly associated with homicide in the univariate analysis, but only loss of contact remained an independent predictor in the final model. However, both are important as markers of non-receipt of care and are discussed in the final subsection of this paper.

Comparable data were unavailable for the patients with schizophrenia who had no history of admission (*n* < 5). Persons who were diagnosed with schizophrenia post-offence, by the psychiatric report author, were not included in the patient case series. NCISH has previously reported that 10% of perpetrators were described in psychiatric reports as being mentally ill at the time of their offence, 4% of whom were thought to have schizophrenia.^14^ In contrast with existing literature,^12^ we found that experiencing delusions and/or hallucinations at the time of the offence appeared to be protective for homicide. This finding may be confounded by the methodological constraints of the control patients being from a data-set of current/recent hospital in-patients. Risk of committing homicide was elevated among patients from BAME groups compared with the White British reference group. The fact that a relative preponderance of the homicide perpetrators from BAME groups resided in the most deprived localities indicates that the elevated homicide perpetration risk in that group could be attenuated, and plausibly entirely explained, by the residual confounding influence of individual- and area-level deprivation. However, we could not assess either of these potential confounding influences. Information on socioeconomic position was unavailable, and area-level deprivation was available via linkage to the PNC only for individuals who had committed homicide. It was unavailable for the control patients.

### Implications for clinical practice and health services

This study adds to the evidence for the relationship between schizophrenia and serious violence. It has been reported previously that much of the elevated risk among these individuals is explained by comorbid substance misuse rather than their mental illness^27,31^ and our findings appear to support this notion. In this study, features of mental healthcare were also associated with homicide, the findings suggesting that maintaining routine treatment and regular contact and avoiding non-adherence to medication and loss of contact are protective. In this clinical population it was exceptionally rare for patient homicide to occur without comorbidity or problems in delivering standard clinical care.

Prevention of serious violence in schizophrenia should therefore focus on addressing comorbidities and maintaining treatment and service contact. This requires collaborative working between mental health and substance misuse services and the introduction and evaluation of models of intensive support from community mental health teams. Maintaining good levels of engagement also requires an understanding of the patient perspective on care and treatment and respect for autonomous decision-making. Furthermore, mental health services should consider the interplay between clinical risk factors and the social environment, recognising that patients who live in deprived areas may need additional support to address substance misuse and maintain contact with services.

## Data Availability

All authors had full access to all the data in the study and take responsibility for the integrity of the data and the accuracy of data analysis; access to the data is currently ongoing. Data from this study cannot be shared because of information governance restrictions in place to protect confidentiality. Access to data can be requested via application to the Healthcare Quality Improvement Partnership (www.hqip.org.uk/national-programmes/accessing-ncapop-data/).
